# Implementing a clinical scientist-led screening clinic for hypertrophic and dilated cardiomyopathies

**DOI:** 10.1186/s44156-024-00045-0

**Published:** 2024-04-17

**Authors:** Jane Draper, Rachel Bastiaenen, Gerald Carr-White, Teofila Bueser, Jessica Webb, Colin Evans, Soraya Nuthoo, Nabeel Sheikh

**Affiliations:** 1 Guy’s and St. Thomas’ NHS Foundation Trust, St. Thomas’ Hospital, Westminster Bridge Road, London, SE1 7EH UK; 2 King’s College London, Faculty of Life Sciences and Medicine, St. Thomas’ Hospital, St. Thomas’ Campus, Westminster Bridge Road, London, SE1 7EH UK; 3 King’s College Hospital NHS Foundation Trust, Denmark Hill, London, SE5 9RS UK

**Keywords:** Cardiomyopathy, Screening, Electrocardiography, Echocardiography

## Abstract

**Background:**

The burden of screening for inherited cardiac conditions on health services grows ever larger, with each new diagnosis necessitating screening of additional family members. Screening these usually asymptomatic, low-risk individuals is currently performed by consultant cardiologists, consuming vital clinic resources that could otherwise be diverted to sicker patients requiring specialist consultant input. Clinical scientists now constitute a highly skilled and often underutilised group of individuals with training in areas such as clinical evaluation, 12-lead electrocardiography (ECG) interpretation, and echocardiography. These skills place them in a unique position to offer a full screening evaluation in a single consultation. The aim of this study was to implement and evaluate a novel clinical scientist-led screening clinic for first-degree relatives of patients with hypertrophic cardiomyopathy (HCM) and dilated cardiomyopathy (DCM). The clinical scientist-led screening clinic was established at a London tertiary centre to allow review of asymptomatic, first-degree relatives of patients with a confirmed diagnosis of HCM or DCM, independent of a cardiology consultant. Patients were evaluated with history, examination, ECG, and echocardiography, with further investigations if deemed necessary. A retrospective review was performed of the first 200 patients seen in the clinic.

**Results:**

Of the 200 individuals reviewed between September 2019 and July 2022, 99 had a proband with HCM and 101 a proband with DCM. Overall, 169 individuals (85%) revealed normal screenings and were discharged. Thirty-one individuals (15.5%), all asymptomatic, revealed ECG changes and/or significant echocardiographic findings. Of these, 21 individuals (10.5% of the total cohort) were subsequently diagnosed with a cardiomyopathy or early phenotypic changes consistent with a cardiomyopathy (11 with HCM and 10 with DCM). These individuals were referred on to an inherited cardiac conditions consultant clinic for regular follow-up. Overall, 179 consultant clinic appointments were saved which could instead be allocated to patients requiring specialist consultant input.

**Conclusions:**

This is the first description of a clinical scientist-led screening clinic for first-degree relatives of patients with HCM and DCM. The findings demonstrate that implementation of such a service into routine clinical practice is feasible, effective, safe, and can free up capacity in consultant clinics for patients requiring specialist input.

## Introduction

Hypertrophic cardiomyopathy (HCM) and dilated cardiomyopathy (DCM) are the two most common inherited cardiac conditions (ICC), with an estimated prevalence of between 1 in 250–500 in the general population [1–5]. Of DCM cases, approximately 20–30% are familial, with a presumed or confirmed genetic aetiology [5, 6]. Both HCM and familial DCM are usually inherited in an autosomal dominant pattern, with first-degree relatives of affected individuals having a 50% chance of inheriting the disease [4, 7, 8]. Given the benefits of early identification but the variable, age-related penetrance of both conditions, international scientific organisations currently recommend periodic screening of all first-degree relatives of affected patients with history, examination, a 12-lead electrocardiogram (ECG), and transthoracic echocardiography [4, 7, 8]. 

Current estimates from National Health Service (NHS) England suggest a combined prevalence of 340,000 for all ICC in the UK, with a prevalence of 121,960 for HCM and at least 5,565 for familial DCM [9]. Providing a regular, periodic screening service to all first-degree relatives of affected individuals represents a significant burden to health services, considering that 18% of UK families have 4–5 members [10], and that each new diagnosis made through screening may necessitate the screening of several additional family members.

The current screening strategy for ICC recommended by NHS England is direct referral to general cardiology or specialist ICC services for evaluation by a consultant cardiologist or appropriately trained clinical nurse specialist (CNS) [9]. However, such services often have significant waiting lists, leading to delays for patients’ relatives and resultant stress and anxiety, particularly if the referral is triggered by the death of a family member. Furthermore, accommodating asymptomatic, low-risk screening patients within specialist consultant-led or CNS clinics results in high-risk referrals and patients with a confirmed diagnosis and/or symptoms who require specialist input waiting longer for their urgent care.

To address such demands, the NHS Long Term Plan [11] acknowledges the need for new ways of working. Specialist skills in performing and interpreting ECGs and echocardiograms are well established within the traditional roles of cardiac physiologists. The Modernising Scientific Careers [12] pathway has fuelled the evolution of this group of professionals to that of clinical scientist [13], extending their scope of practice to create a uniquely trained and now registered workforce. Furthermore, clinical scientists specialising in echocardiography who also hold advanced practice skills of ECG interpretation, clinical history taking, and clinical examination can offer a comprehensive review of patients together with diagnostics in a single clinical consultation (the “one-stop shop” model). Such clinics have already proven successful in other areas of cardiology such as valvular heart disease, are highly efficient, and provide novel solutions for alleviating pressures on existing patient pathways [14, 15]. 

The aim of this study was to describe and evaluate the impact of implementing a clinical scientist-led service for family screening of asymptomatic first-degree relatives of patients with HCM or DCM within our institution.

## Methods

### Setting

Guy’s and St Thomas’ NHS Foundation Trust runs busy specialist cardiomyopathy clinics in London, UK, as part of its ICC service. These clinics are run by eight ICC consultants and serve the local community in the London boroughs of Lambeth, Southwark, and Lewisham. The service also receives referrals from across London, the entire southeast of England, and the rest of the United Kingdom (UK). A recent audit of our ICC service estimated approximately 10,000 patient contacts per year. It is within this framework that the clinical scientist-led screening clinic was established, led by a single clinical scientist (JD) at the time of writing. The data for this article can be shared on reasonable request to the corresponding author.

### The clinical scientist-led screening clinic

#### Clinical scientist training

Registration with the Health and Care Professions Council is required for clinical scientist status. The clinical scientist leading our screening clinic achieved registration via an equivalence process through the Academy of Healthcare Science where she demonstrated adherence to good scientific practice and evidence of providing safe, knowledgeable, and professional services within healthcare sciences, equivalent to a three-year programme of work-based learning, supported by a university. Additionally, prior to establishing the clinic, the clinical scientist already held accreditation in transthoracic echocardiography through the British Society of Echocardiography and a Clinical Assessment for Healthcare Scientists Master of Science qualification. Skills in ECG interpretation were obtained from prior cardiac physiologist training. Specific training for the clinical scientist-led screening clinic was provided by a period of one-to-one training with specialist ICC consultants to achieve internal competencies for safe, independent review of patients. This initially involved joint review of patients with an ICC consultant, but subsequently progressed to discussion and overview of patients seen independently by the clinical scientist to ensure consultant agreement with the assessment and plan. This training took place in outpatient clinics and inpatient ward settings and exceeded 50 clinical hours of training over a 4-month period.

#### Scope of the clinic scientist-led screening clinic

**Fig. 1:**
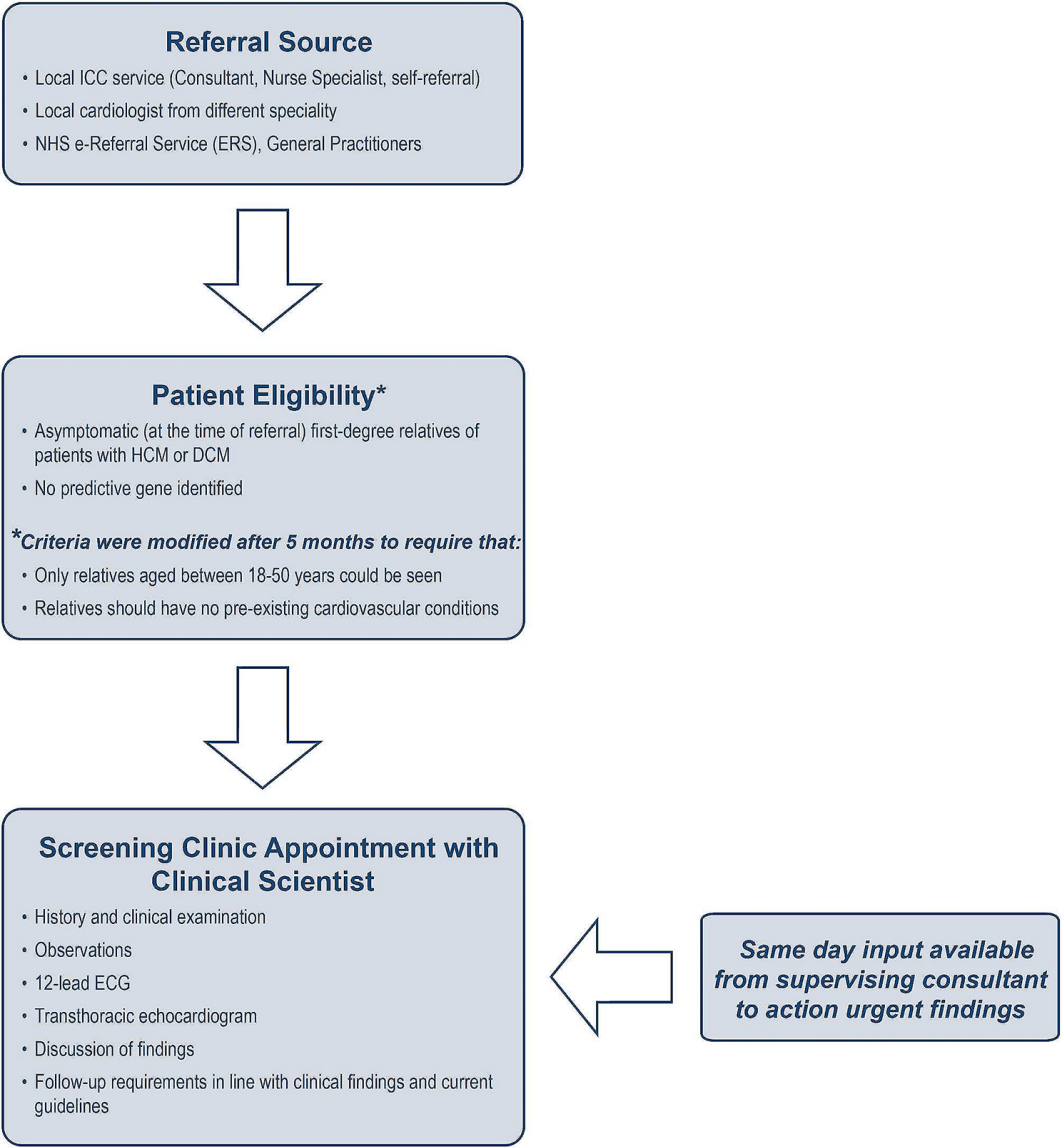
Patient eligibility and pathway through clinical scientist-led inherited Cardiac conditions screening clinic. DCM, dilated cardiomyopathy; ECG, electrocardiography; HCM, hypertrophic cardiomyopathy; TTE, transthoracic echocardiography

The clinical scientist-led screening clinic was established to allow independent review of asymptomatic, first-degree relatives of patients with a confirmed diagnosis of HCM or DCM, without patients needing an appointment with an ICC consultant. At the time of writing, a single clinical scientist ran the clinic and evaluated 4 patients per clinic, with 4 clinics taking place per month. Referrals to the service originated from within our own ICC service, from other cardiologists (either at Guy’s and St Thomas’ NHS Foundation Trust or another institution), from general practitioners, and directly from patients via self-referral forms issued by our ICC service. The patient pathway is summarised in Fig. 1. The clinic was established to run alongside a cardiologist’s ICC clinic so support could be provided to the clinical scientist should this be required.

#### Eligibility criteria

Patient eligibility criteria for the clinical scientist-led clinic are summarised in Fig. 1. Although the aim of the clinic was to evaluate relatives without symptoms (as detailed on the referral letter) at the time of referral, a proportion of individuals subsequently reported symptoms at their clinic visit, either because they had developed symptoms since being referred or because they had failed to mention symptoms at the time of their referral. Genotype-positive, phenotype-negative relatives were excluded from the clinic as it was felt that these individuals would need long-term follow-up and may potentially require further investigations beyond an ECG and echocardiogram to completely exclude a phenotype; they would therefore not fit the “one-stop shop” model the clinic was aspiring to emulate. The criteria were modified after 5 months to exclude patients below the age of 18, above the age of 50, and those with pre-existing cardiovascular disease. This decision was taken after findings from continual audit, which highlighted older patients or those with known comorbidities, particularly hypertension, often required subsequent input by a cardiologist; it was therefore felt that such patients would be better served in a consultant clinic.

### Clinical evaluation

All patients were evaluated with history and physical examination of their cardiac and respiratory systems and were then investigated with an ECG and 3-dimensional transthoracic echocardiogram.

#### 12-Lead electrocardiography

Standard 12-lead electrocardiography was performed using a Spacelabs CardioExpress SL18A resting ECG machine (Spacelabs Healthcare, Snoqualmie, Washington, USA) with individuals in the supine position. The definition of specific ECG abnormalities is summarised in Table 1.

**Table 1:** Definition of abnormal ECG findings

ECG abnormality	Definition
Left atrial enlargement	Negative portion of the P wave in lead V1 ≥ 0.1 mV in depth and ≥ 40 msec in duration
Right atrial enlargement	P-wave amplitude ≥ 0.25 mV in leads II, III or aVF
Left QRS-axis deviation	-30^O^ to -90^O^
Right QRS-axis deviation	> 115^O^
Right ventricular hypertrophy	Sum of R wave in V1 and S wave in V5 or V6 ≥ 10.5 mm
Complete LBBB	QRS ≥ 120 msec predominantly negative QRS complex in lead V1 (QS or rS), and upright monophasic R wave in leads I & V6
Complete RBBB	RSR’ pattern in anterior precordial leads with QRS duration ≥ 120 msec
Intraventricular conduction delay	Any QRS duration > 120 msec including RBBB and LBBB
Pathological Q-wave	>-0.4 mV in any lead except III, aVR or a Q/R ratio ≥ 0.25
T-wave inversion	≥-0.1 mV in ≥ 2 contiguous leads other than in leads V1, aVR and III
ST-segment depression	≥ 0.5 mm deep in ≥ 2 leads
Ventricular pre-excitation	PR interval < 120 msec with delta wave

LBBB: left bundle branch block; RBBB: right bundle branch block

#### Transthoracic echocardiography

Standard transthoracic views of the heart were obtained using a General Electric (GE) Vivid E95 ultrasound machine and 4 V-D phased array transducer (GE Healthcare, Chicago, Illinois, USA). Full transthoracic echocardiography dataset images were acquired and analysed in accordance with British Society of Echocardiography guidelines [16]. Chamber dimensions were measured in the parasternal long-axis view. Left ventricular (LV) wall thickness measurements were made in the parasternal long-axis and short-axis views. Single best evaluation of LV ejection fraction (EF) was calculated by (preference in descending order): (i) 3D volumes; (ii) Simpson’s Biplane method; and (iii) visual estimate, as image quality allowed [17]. Global deformation of the left ventricle was assessed with strain rate imaging. Indices of diastolic function were assessed in the apical 4-chamber view with pulsed-wave Doppler across the mitral valve and tissue Doppler imaging of the septal and lateral mitral valve annulus [18]. Comprehensive evaluation of valve function was performed using colour and spectral Doppler.

### Analysis of clinical scientist-led screening clinic data

Individuals who met eligibility criteria (Fig. 1) at the time of referral were seen in the clinical scientist-led screening clinic. A retrospective review of outcomes for the first 200 patients evaluated within the clinic was performed. Demographics, reported symptoms, ECG findings, and echocardiographic findings were reviewed. Abnormal screenings were defined as abnormalities found on the 12-lead ECG (Table 1), the echocardiogram, or both; or symptoms causing concern to the clinical scientist, as described in international guidelines for DCM and HCM [4, 19]. The number of consultant clinic slots saved, additional investigations, and referral to consultant services were also evaluated.

### Statistical analysis

Continuous variables are represented as mean ± standard deviation and categorical variables as number (percentage) of individuals.

## Results

Most patients evaluated in the screening clinic were referred through self-referral forms provided to family members of patients with a known diagnosis of HCM or DCM (*n* = 116). Of the remainder, 66 were referred from ICC services, 16 from local general practitioners, and 2 from non-ICC cardiologists.

Between September 2019 and July 2022 (with periods of cessation during Covid-19 lockdowns), 200 individuals were evaluated in the clinical scientist-led screening clinic. Of these, 101 had a proband with HCM (mean age 36 ± 14 years; 53.5% male) and 99 had a proband with DCM (mean age 34 ± 12 years; 54.5% female). Of those individuals screened with a proband with HCM, 12 were of Afro-Caribbean ethnicity, 16 of south Asian ethnicity, and 73 were Caucasian. Of those individuals screened with a proband with DCM, 3 were of Afro-Caribbean ethnicity, 1 of south Asian ethnicity, and 95 were Caucasian. Overall, 169 (84.5%) individuals revealed normal cardiac screenings and were reassured and discharged, with recommendation for repeat screening at intervals appropriate for age or if symptoms of concern developed before the next screening was due. Thirty-one patients (15.5%) were found to have ECG changes and/or significant echocardiographic findings. Of these, 21 patients (10.5% of the total cohort) were subsequently diagnosed with a cardiomyopathy or early phenotypic changes consistent with a cardiomyopathy and referred on to an ICC consultant clinic for regular follow-up.

### Screening outcomes

**Fig. 2:**
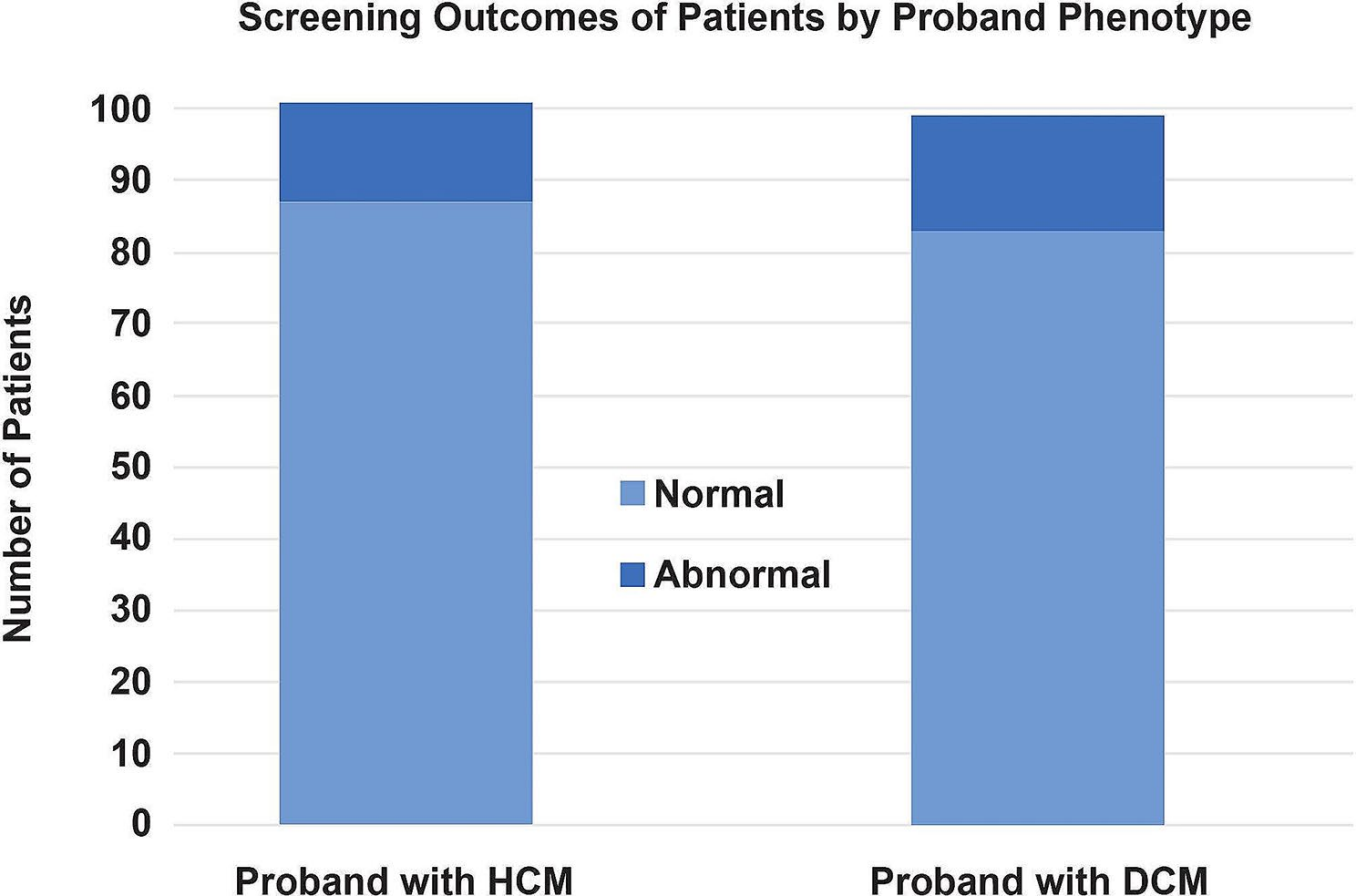
Screening outcomes of patients by proband phenotype. DCM, dilated cardiomyopathy; HCM, hypertrophic cardiomyopathy

Figure 2 summarises the screening outcomes by proband group. The most common symptoms reported by patients were palpitation (*n* = 13; 6.5%) and non-cardiac sounding chest pain (*n* = 8; 4%). A further 4 patients (2%) reported breathlessness, and 3 patients (1.5%) a history of syncope consistent with a vasovagal episode. Twenty-four-hour ambulatory ECG monitors were requested for 13 patients; of these, 12 did not show any significant arrhythmia. One monitor reported a 1% burden of ventricular ectopics, and the patient was referred on to the consultant-led services. On clinical examination, 15 patients (7.5%) were found to have hypertension. Of these, 5 were referred on for 24-hour ambulatory blood pressure monitors and recommendations made to their general practitioners for medication optimisation, as appropriate. All patients found to have abnormal screenings were asymptomatic and had normal cardiac and respiratory examinations.

#### Individuals with a HCM proband

Of the 101 individuals screened with a HCM proband, abnormalities on the ECG and/or echocardiogram were identified in 15 (14.9%), who were subsequently recommended a cardiac MRI (CMR) for further evaluation. Of these 15 individuals, one declined further investigation and 11 (78.6%) were found to have HCM or early phenotypic features suggestive of HCM. These 11 patients were referred onto an ICC consultant and remain under long-term follow-up. The remaining 3 individuals had a normal CMR and were reassured and discharged. Table 2 summarises the abnormalities found during screening and on CMR in the 11 individuals subsequently diagnosed with HCM or identified to have early phenotypic changes.

**Table 2:** Abnormalities found during screening and subsequent cardiac MRI in the 11 individuals diagnosed with HCM.

Proband diagnosis	Age	Gender	Ethnicity	ECG abnormality	Echo abnormality	Cardiac MRI
HCM	23	F	Afro-Caribbean	LVH, widespread T-wave inversion	IVS 18 mm, SAM, LVOTO	Under Cardiology Follow up elsewhere
HCM	30	M	White	LVH, Deep T-wave inversion V1-V2	IVS 13 mm	Mildly dilated left ventricle with mildly impaired global LV systolic function at rest (LVEF 51%). Eccentric hypertrophy with increased indexed LV mass. Small crypts in the inferior LV wall
HCM	77	M	White	Nil	IVS 14 mm	Normal LV end-diastolic volume and systolic function (LVEF 58%). Increased asymmetric septal LVH (15 mm in the basal anteroseptum). Prominent insertion point fibrosis with less dense basal-to-mid septal fibrosis and further focus of mid-wall fibrosis in the basal inferoseptum
HCM (apical)	50	M	Asian	Nil	Suboptimal echo images with apex not clearly visualised	Normal indexed LV end-diastolic volume with hyperdynamic function (LVEF 75%). LVH of 12 mm in septum and increased index LV mass. Apical cavity obliteration during systole with apically displaced papillary muscles
HCM	54	M	White	Frequent ectopy	Dilated LV cavity with severely impaired function (LVEF 21%)	Patient under local cardiology follow-up
HCM	44	M	Asian	Nil	Reduced GLS − 13%	Normal indexed LV end-diastolic volume and function (LVEF 66%). Mild asymmetric septal LVH of 13–14 mm in the basal-to-mid septum
HCM	41	M	Asian	Nil	Reduced GLS − 11%	Normal indexed LV end-diastolic volume, function (LVEF 59%), and wall thickness but subtle findings including abnormal septal convexity and a single inferior wall crypt
HCM	30	M	Asian	T-wave inversion in leads III and aVF	Nil	Normal indexed LV end-diastolic volume and systolic function (LVEF 57%), but with prominent apical trabeculation and apically displaced papillary muscles, elongation of the anterior mitral valve leaflet and insertion point fibrosis. No overt LVH.
HCM	48	M	Afro-Caribbean	High take off V1-V4	IVS 14 mm	Normal indexed LV end-diastolic volume and systolic function (LVEF 58%). Increased LV wall thickness of 12 mm. Inferior wall crypt and elongated anterior mitral valve leaflet.
HCM	77	F	Afro-Caribbean	T-wave inversion in leads I, aVL	IVS 16 mm	Normal indexed LV end-diastolic volume and systolic function (LVEF 58%). and systolic function (LVEF 62%). Asymmetric septal LVH with maximal wall thickness of 16–17 mm in the basal anterior wall, partial chordal SAM, and apical displacement of hypertrophied papillary muscles.
HCM	52	M	White	Flattened T-waves V4-V5	Suspicion of apical hypertrophy	Normal indexed LV end-diastolic volume and wall thickness with mildly impaired systolic function (EF 51%). Accessory antero-apical papillary muscle, small crypt in the inferior wall, and sub-epicardial / mid-wall fibrosis of the basal anterolateral wall and entire inferolateral and inferior walls.

F: female; GLS: Global longitudinal strain; HCM: Hypertrophic cardiomyopathy; IVS: interventricular septum; LV: left ventricular; LVEF: left ventricular ejection fraction; LVH: Left ventricular hypertrophy; LVOTO: left ventricular outflow tract obstruction; M: male; SAM: systolic anterior motion of the mitral valve leaflets

#### Individuals with a DCM proband

**Fig. 3:**
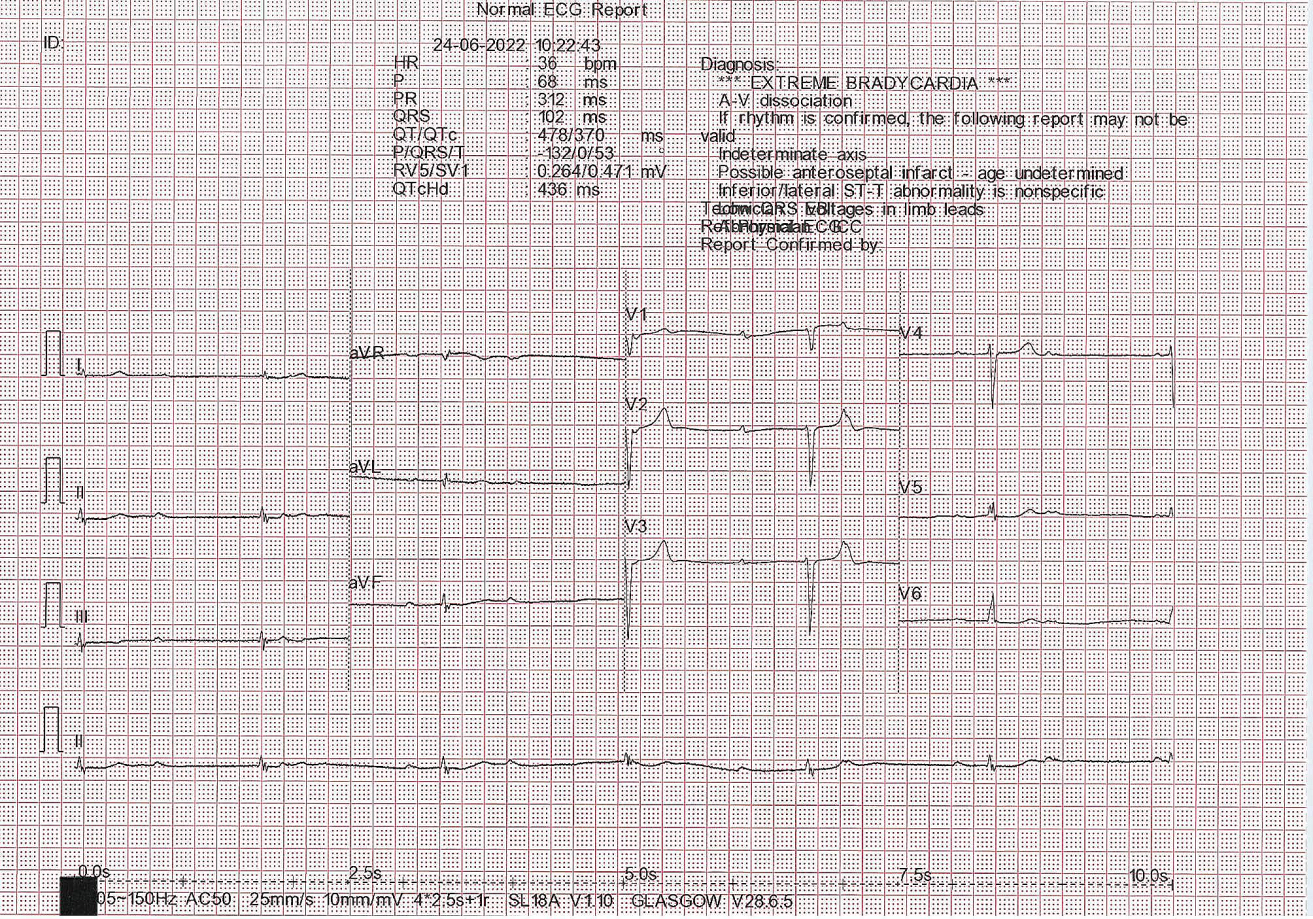
ECG from a 36-year-old man attending the clinical scientist-led screening clinic demonstrating complete heart block

Of the 99 individuals screened with a DCM proband, 5 were identified as having a family history of possible arrhythmogenic cardiomyopathy rather than DCM during the clinical scientist-led clinic. These 5 individuals were therefore considered inappropriate referrals and referred on to an ICC consultant with further diagnostic tests completed. Of the remaining 94 individuals, abnormalities on the ECG and/or echocardiogram were identified in 16 (17.0%), who were subsequently recommended a CMR for further evaluation. Of these 16 individuals, 2 declined further investigations, 4 revealed normal CMR findings and were reassured and discharged, and 10 demonstrated findings consistent with DCM or early phenotypic features of DCM. These 10 patients were referred onto an ICC consultant and remain under long-term follow-up. Table 3 summarises the findings identified during screening and subsequent CMR in these 10 individuals. Of note, 1 patient was admitted from clinic after the screening ECG demonstrated complete heart block (Fig. 3) with a dilated left ventricle and mildly reduced EF. An inpatient CMR confirmed a dilated LV with mildly reduced global LV systolic function (EF 53%), normal right ventricular size and function, and basal septal mid-wall linear fibrosis with inferior insertion point fibrosis. A biventricular implantable cardioverter-defibrillator was inserted, and subsequent genetic testing revealed a pathogenic variant in the Lamin A/C gene. Two months later, the patient received appropriate anti-tachycardia pacing for fast ventricular tachycardia.

**Table 3:** Abnormalities found during screening and subsequent cardiac MRI in the 10 individuals diagnosed with DCM.

Proband Diagnosis	Age	Gender	Ethnicity	ECG abnormality	Echo Abnormality	Cardiac MRI
DCM	35	M	White	Complete heart block	Dilated LV cavity, LVEF 53%	Increased indexed LV end-diastolic volume with mildly reduced systolic function (LVEF 53%). Basal septal mid-wall linear and RV inferior insertion point fibrosis
DCM	23	M	White	Nil	LVEF 48%, GLS − 14.3%	Normal indexed LV end-diastolic volume with mildly reduced systolic function (LVEF 53%); elongated anterior mitral leaflet with papillary muscle variants and single myocardial crypt
DCM	41	M	White	Voltage criteria for LVH	Borderline LV cavity dimensions Low-normal LVEF 53%	Increased indexed LV end-diastolic volume with mildly reduced systolic function (LVEF 54%)
DCM	32	M	White	Nil	Dilated LV volumes	Increased indexed LV end-diastolic volume with mildly impaired systolic function (LVEF 50%). Normal RV size and function.
DCM	37	M	Afro-Caribbean	Nil	LVEF 52%, GLS − 15%	Normal indexed LV end-diastolic volume with moderately impaired LV systolic function (LVEF 43%). Normal indexed RV volume with mild RV impairment (EF 40%).
DCM	26	F	White	Nil	LVEF 47%	Normal Indexed LV end-diastolic volume with moderately impaired global systolic function (LVEF 43%)
DCM	55	M	White	Nil	LVEF 42%, GLS − 15%	Normal Indexed LV end-diastolic volume with mildly impaired global systolic function (LVEF 50%). Basal-to-mid mid-wall septal fibrosis; epicardial and mid-wall fibrosis of the basal-to-mid lateral and inferior walls
DCM	27	M	White	Nil	Dilated LV volumes with borderline EF (53%)	Normal indexed LV end-diastolic volume with moderately impaired global systolic function (LVEF 43%)
DCM	30	F	White	T-wave inversion V1-V6	LVEF 50%	Normal LV with borderline reduced systolic function (LVEF 55%) and increased trabeculation of the LV anterior wall
DCM	46	M	White	Atrial ectopy	LVEF 50%, GLS − 16%	Unable to tolerate full cardiac MRI scan due to claustrophobia

DCM: dilated cardiomyopathy; F: female; GLS: global longitudinal strain; IVS: interventricular septum; LV: left ventricular; LVEF: left ventricular ejection fraction; LVH: left ventricular hypertrophy; M: male

### Comparison to consultant clinics

We compared the results from our clinical scientist-led screening clinic to recent data we have from screenings performed in our consultant-led clinics. Over the period January 2023 to January 2024, fifty individuals who did not meet eligibility criteria for the clinical scientist-led clinic were screened for either HCM (*n* = 26; 52%) or DCM (*n* = 24; 48%) in a consultant clinic. Of the HCM cohort, 17 individuals (65.4%) were cleared and discharged after screening and 9 (34.6%) were referred for a CMR. Of the 9 referred for a CMR, 4 individuals (44.4%) were cleared after the scan and 5 individuals (55.6%) remain under long-term follow-up for borderline or suspicious changes. Of the DCM cohort, 19 individuals (79.2%) were cleared and discharged after screening and 5 (20.8%) were referred for a CMR. Of the 5 referred for a CMR, one individual (20%) was diagnosed with DCM and 4 individuals (80%) remain under long-term follow-up for borderline or suspicious changes. Overall, 72% of relatives were discharged directly after screening in a consultant clinic compared to 84% after screening in the clinical scientist-led clinic, meaning that almost one-third of patients seen in a consultant clinic required further investigations compared to just 15% in the clinical scientist-led clinic. Furthermore, 20% of patients screened in a consultant clinic required on-going, long-term surveillance, compared to almost half that proportion (10.8%) screened in the clinical scientist-led clinic.

### Cost and resource implications

The main cost-saving achieved from the clinical scientist-led clinic was consultant time, given that the clinical scientist staffing salary at Agenda for Change Band 8a (£50,952 to £57,349 per year, depending on experience [20]) is lower than that of a consultant cardiologist’s basic salary (£88,364 to £119,133 per year on the 2003 contract [20]). Furthermore, a transthoracic echocardiogram is incorporated within the clinic structure of the clinical scientist-led clinic, saving capacity in the echocardiography department.

## Discussion

To date, this is the first report of a specialist clinical scientist-led family screening clinic for HCM and DCM in the UK. The usual pathway for screening first-degree relatives of patients with these conditions would involve referral to a consultant cardiologist or CNS. NHS England recognise that ICC services are unable to meet ever-increasing demands [9], and the data presented here demonstrates the benefit of a clinical scientist-led service to relieve pressures on consultant and CNS clinic appointments and improve resource utilization; this service model removes younger, asymptomatic family members from specialist waiting lists. Indeed, 84% of our cohort did not require consultant review or input, and 179 consultant clinic appointments were saved which could instead be allocated to patients with confirmed diagnoses or those at higher risk requiring specialist consultant input. Our results also illustrate that compared to our consultant clinics, more individuals seen in the clinical scientist-led clinic were cleared and discharged after the initial screening (84% vs. 72%), and fewer required further investigations (16% vs. 28%) and/or long-term follow-up (10.8% versus 20%). This highlights the need for robust inclusion and exclusion criteria and accurate referral triage so that patients with, for example, symptoms or a positive gene test, in whom the pre-test probability of disease is higher, are seen by the most appropriate specialist from the outset, avoiding duplication and unnecessary resource utilisation.

Screening first-degree relatives of patients with HCM or DCM is important for identification of asymptomatic individuals harbouring unrecognised disease. This is highlighted by our data, which demonstrates that screened individuals subsequently diagnosed with disease were well and asymptomatic at the time of referral. Given that this patient cohort are mostly asymptomatic, they may not be prioritised for review and encounter long waits when first referred. A service provision model as described here provides an alternative pathway for these individuals to be seen, reducing waiting times and likely alleviating stress and anxiety that long waiting times may cause. Such a clinic has advantages over consultant and CNS-led clinics as it avoids patients needing to be allocated multiple, separate resources in the form of a nurse, ECG technician, and echocardiographer. As these roles are combined into one, it makes a clinical scientist-led service highly cost and resource effective. Such a clinic also has advantages over open access diagnostic referrals from general practitioners for stand-alone ECGs and echocardiograms, providing a more comprehensive review and same-day interpretation of test results within the framework of a multidisciplinary service that has readily available access to ICC consultants and specialist genetic nurses for immediate input and patient support, as required.

Following on from the NHS Long Term Plan (previously known as the 10-year Plan) [21], NHS England commissioned a review of diagnostic services in England and Wales [22] which advocates for investment in equipment, facilities, and workforce in order to transform these currently overburdened services. The review also calls for different ways of working, promotes a “one-stop shop” model for patient care, and identifies diagnostic hubs as ways of bringing care closer to patients. The service we have described here has the potential to meet all these requirements. Advanced practitioner roles help increase capacity, reduce waiting times [23], and can potentially be set up away from tertiary centres in district general hospitals, reducing traveling and increasing convenience for patients. In addition, developing and expanding roles for clinical scientists with advanced practice offers the opportunity for career progression, which may in turn help increase staff retention and attract new individuals into a workforce group with a recognised national shortage.

The potential of clinical scientists is often under recognized and poorly understood, but as a staff group these individuals comprise a highly skilled workforce who are already involved in many aspects of patient care. NHS England advocate the need for a multidisciplinary approach to ICC services. Traditionally, this has been limited to consultant cardiologists and specialist nurses. Given that screening guidelines for cardiomyopathies encompass ECG and echocardiography at the centre of the diagnostic pathway, harnessing the expertise of clinical scientists who routinely deliver and interpret these diagnostic modalities can only be of benefit. Other specialities where clinical scientist-led services have been successful have robust and defined levels of experience and training. For the clinic described here, that includes extensive experience in echocardiogram and ECG interpretation, professional standards to achieve registration with the Health and Care Professions Council, and most importantly, one-on-one clinical time spent with a supervising consultant to ensure appropriate skills and training.

### Limitations

This study is limited by being single centre, retrospective, and observational. It is not clear how well this service model would translate to smaller centres where the role of the clinical scientist is not well promoted, or in centres without designated specialist ICC services. Further work is also required to assess patient satisfaction, including patient’s views on being seen by a clinical scientist rather than a physician, although publications looking at other non-physician run services suggest that patients are comfortable with being reviewed by healthcare professionals that are not medical doctors [24–26].

## Conclusion

This study describes the first clinical scientist-led screening clinic for first-degree relatives of patients with HCM and familial DCM. Our findings demonstrate that implementing this service into routine clinical practice is feasible, can help save appointments with specialist ICC consultants, and provides efficient and safe review of patients within this defined population.

## Data Availability

The datasets generated and/or analysed during the current study are not publicly available due to patient confidentiality but are available from the corresponding author on reasonable request.

## Abbreviations

CMR: Cardiac MRI
DCM: Dilated cardiomyopathy.
ECG: 12-lead electrocardiogram.
EF: Ejection fraction.
HCM: Hypertrophic cardiomyopathy.
ICC: Inherited cardiac conditions.
LV: Left ventricular.
NHS: National health service.
UK: United Kingdom.
